# *TP53* mutation status divides myelodysplastic syndromes with complex karyotypes into distinct prognostic subgroups

**DOI:** 10.1038/s41375-018-0351-2

**Published:** 2019-01-11

**Authors:** Detlef Haase, Kristen E. Stevenson, Donna Neuberg, Jaroslaw P. Maciejewski, Aziz Nazha, Mikkael A. Sekeres, Benjamin L. Ebert, Guillermo Garcia-Manero, Claudia Haferlach, Torsten Haferlach, Wolfgang Kern, Seishi Ogawa, Yasunobu Nagata, Kenichi Yoshida, Timothy A. Graubert, Matthew J. Walter, Alan F. List, Rami S. Komrokji, Eric Padron, David Sallman, Elli Papaemmanuil, Peter J. Campbell, Michael R. Savona, Adam Seegmiller, Lionel Adès, Pierre Fenaux, Lee-Yung Shih, David Bowen, Michael J. Groves, Sudhir Tauro, Michaela Fontenay, Olivier Kosmider, Michal Bar-Natan, David Steensma, Richard Stone, Michael Heuser, Felicitas Thol, Mario Cazzola, Luca Malcovati, Aly Karsan, Christina Ganster, Eva Hellström-Lindberg, Jacqueline Boultwood, Andrea Pellagatti, Valeria Santini, Lynn Quek, Paresh Vyas, Heinz Tüchler, Peter L. Greenberg, Rafael Bejar, for the International Working Group for MDS Molecular Prognostic Committee

**Affiliations:** 1 University Medical Center, Georg- August-University, Goettingen, Germany; 2 Dana-Farber Cancer Institute, Boston, MA USA; 3 Cleveland Clinic Taussig Cancer Center, Cleveland, OH USA; 4 University of Texas MD Anderson Cancer Center, Houston, TX USA; 5 MLL Munich Leukemia Laboratory, Munich, Germany; 6 Kyoto University, Kyoto, Japan; 7 Massachusetts General Hospital Cancer Center, Boston, MA USA; 8 Washington University School of Medicine, St. Louis, MO USA; 9 H. Lee Moffitt Cancer Center and Research Institute, Tampa Bay, FL USA; 10 Memorial Sloan Kettering Cancer Center, New York, NY USA; 11 Wellcome Trust Sanger Institute, Cambridge, UK; 12 Vanderbilt-Ingram Cancer Center, Nashville, TN USA; 13 Hôpital St Louis, Assistance Publique-Hôpitaux de Paris and Paris Diderot University, Paris, France; 14 Chang Gung Memorial Hospital and Chang Gung University, Taoyuan, Taiwan; 15 St. James’s Institute of Oncology, Leeds Teaching Hospitals, Leeds, UK; 16 University of Dundee, Ninewells Hospital, Dundee, UK; 17 Université Paris Descartes, Hopital Cochin Assistance Publique-Hopitaux de Paris, Paris, France; 18 Tisch Cancer Institute, Icahn School of Medicine at Mount Sinai, New York, NY USA; 19 Hannover Medical School, Hannover, Germany; 20 Fondazione IRCCS Policlinico San Matteo & University of Pavia, Pavia, Italy; 21 University of British Columbia, Vancouver, BC Canada; 22 Karolinska Institutet, Karolinska University Hospital, Stockholm, Sweden; 23 Radcliffe Department of Medicine, University of Oxford, Oxford, UK; 24 MDS Unit, AOU Careggi, University of Florence, Florence, Italy; 25 MRC Molecular Hematology Unit, WIMM University of Oxford, Oxford, UK; 26 Haematology Theme Oxford Biomedical Research Centre and Department of Hematology, Oxford University Hospitals NHS Foundation Trust, Oxford, UK; 27 Ludwig-Boltzmann Institute for Leukemia Research, Vienna, Austria; 28 Stanford University Cancer Institute, Stanford, CA USA; 29 UC San Diego Moores Cancer Center, La Jolla, CA USA

**Keywords:** Myelodysplastic syndrome, Myelodysplastic syndrome, Cancer genomics, Translational research

## Abstract

Risk stratification is critical in the care of patients with myelodysplastic syndromes (MDS). Approximately 10% have a complex karyotype (CK), defined as more than two cytogenetic abnormalities, which is a highly adverse prognostic marker. However, CK-MDS can carry a wide range of chromosomal abnormalities and somatic mutations. To refine risk stratification of CK-MDS patients, we examined data from 359 CK-MDS patients shared by the International Working Group for MDS. Mutations were underrepresented with the exception of *TP53* mutations, identified in 55% of patients. *TP53* mutated patients had even fewer co-mutated genes but were enriched for the del(5q) chromosomal abnormality (*p* < 0.005), monosomal karyotype (*p* < 0.001), and high complexity, defined as more than 4 cytogenetic abnormalities (*p* < 0.001). Monosomal karyotype, high complexity, and *TP53* mutation were individually associated with shorter overall survival, but monosomal status was not significant in a multivariable model. Multivariable survival modeling identified severe anemia (hemoglobin < 8.0 g/dL*), NRAS* mutation, *SF3B1* mutation, *TP53* mutation, elevated blast percentage (>10%), abnormal 3q, abnormal 9, and monosomy 7 as having the greatest survival risk. The poor risk associated with CK-MDS is driven by its association with prognostically adverse *TP53* mutations and can be refined by considering clinical and karyotype features.

## Introduction

Risk stratification is essential in the clinical care of patients with myelodysplastic syndromes (MDS). The predicted prognosis helps physicians select when and how to treat and sets expectations for patients and families. Recurrent cytogenetic abnormalities are powerful predictors of prognosis in MDS and are included in several prognostic scoring systems used in clinical practice [1]. Individual abnormalities can have a wide range of prognostic associations when present in isolation. For example, deletion of chromosome 5q is favorable while loss of chromosome 7 is adverse [2]. In contrast, the presence of three or more chromosomal abnormalities is always considered adverse, regardless of which lesions are present [3, 4]. Prognostic models such as the Revised International Prognostic Scoring System (IPSS-R) assign substantial risk to the 10% of MDS patients with a complex karyotype (CK), defined as three or more somatic chromosomal abnormalities present in a single clone. The IPSS-R considers patients with exactly three abnormalities to have ‘Poor’ cytogenetic risk, while those with four or more abnormalities have ‘Very Poor’ cytogenetic risk, the highest possible risk category, with a score that exceeds that assigned to bone marrow blasts >10% [2, 5]. In fact, the presence of CK excludes most MDS patients from having ‘lower risk’ MDS, as defined by the IPSS-R, in the presence of even one additional risk factor.

While there are no good actors in this traditionally high-risk population, complex karyotype MDS patients represent a heterogeneous group whose overall survival and disease course is affected by factors other than the number of chromosomal abnormalities they carry [3]. The types of abnormalities present, co-occurring somatic mutations, and clinical features all contribute to the actual risk in patients with complex karyotypes. Several groups have examined the prognostic impact of a monosomal karyotype (MK), defined as a complete loss of an autosomal chromosome in the presence of at least one other structural abnormality or additional monosomy, as in practice, most patients with MK also have CK [6]. Parsing complex karyotypes as monosomal can identify MDS patients with even greater risk than predicted by tools like the IPSS-R, although the independent prognostic significance of MK is still debated [7–12]. Other studies have focused on the high frequency of *TP53* mutations in patients with complex karyotypes [13–17]. *TP53* mutations have highly adverse prognostic implications in a wide variety of clinical settings that are independent of other risk factors [18–25]. This is despite their association with adverse clinical features such as increased blast proportion, severe thrombocytopenia, and multiple chromosomal abnormalities [13–15, 21, 26]. The type and abundance of *TP53* mutation in question may further refine its prognostic impact [27–29]. The extent to which *TP53* mutations can modify risk assessment in otherwise higher risk MDS patients with multiple chromosomal abnormalities remains unclear.

To examine the impact of somatic mutations in CK-MDS, the International Working Group (IWG) for MDS Molecular Prognosis Committee collected clinical and mutational information about complex karyotype MDS patients evaluated at 19 centers internationally. We examined risk-associated markers in complex karyotype MDS such as the presence of MK, specific chromosomal lesions, total number of lesions, clinical variables, and the presence of *TP53* mutations to determine which features had independent prognostic value that could be used to better risk stratify patients with complex karyotype MDS.

## Materials and methods

### Patient data collection

Members of the IWG for MDS shared clinical and mutation data on 359 patients with complex karyotypes collected from 19 centers (Supplemental Table 1) some of whom were included in previously published MDS cohorts [13–15, 24, 30]. Patients consented to sample collection, analysis, and clinical annotation at their home institution on protocols approved by local ethics review boards in accordance with the Declaration of Helsinki. All data shared for this study were assigned unique patient identifiers. Anonymized patient data included age, sex, blood counts, bone marrow blast proportion, somatic mutations calls, and conventional G-banded karyotype results. Patients were excluded from further study if they did not meet criteria for a complex karyotype after manual review, had a sequenced sample collected only at the time of stem cell transplantation, or had a diagnosis of acute myeloid leukemia (AML) with ≥30% blasts at the time of sample collection. Patients with oligoblastic AML with up to 29% blasts were included.

### Karyotype review

Every complex karyotype was manually reviewed and parsed independently by RB and DH blinded to the clinical information or *TP53* mutation status associated with the patient. Discrepancies in total numbers of chromosome abnormalities, monosomal status, or the presence of specific abnormalities were resolved jointly by RB and DH. A brief schema with examples describing the approach used to count and identify chromosomal abnormalities can be found in Supplemental Table 2.

### Mutation assessment

Each center performed its own sequencing to interrogate the *TP53* gene, resulting in a call of presence or absence of a *TP53* mutation. This included Sanger sequencing or various forms of next-generation sequencing. Some centers reported only the presence or absence of a *TP53* mutation, while others provided the DNA change, the predicted impact on coding amino acid sequence, and the variant allele fraction. Several centers reported the presence or absence of other mutations from larger panels of myeloid malignancy-associated genes.

### Statistical analysis

Patient characteristics were compared between groups using Fisher's exact test for categorical data and the Wilcoxon rank-sum test for continuous measures. Overall survival (OS) was measured from the time of sample collection for the determination of mutational status to the time of death from any cause. OS curves were constructed using the method of Kaplan and Meier and compared using the log-rank test. OS was evaluated in Cox proportional hazard regression modeling univariately and a stepwise procedure was used to determine a final multivariable model. Patient characteristics (age, sex, bone marrow blast %, hemoglobin, absolute neutrophil count, and platelet count categorized as shown in the patient characteristic table and IPSS-R), karyotypic features (number of abnormalities, monosomal karyotype, abnormal 17, 17p deletion with predicted loss of the *TP53* locus, −7, del(7q), del(5q), abnormal 3q, der(1;7), abnormal 9, −13/13q, −18/18q, −21, +21), and mutational status (including the presence or absence of mutations in *TP53*, *DNMT3A*, *ASXL1*, *TET2*, *U2AF1*, *RUNX1*, *JAK2*, *SF3B1*, *CBL*, *NRAS*, *KRAS*, *EZH2*, *SRSF2*, *IDH1*, and *IDH2*) were included as candidates in the modeling where at each step the variable entry criterion was *p* < 0.20 and variables were retained in the model if *p* < 0.05. Models including IPSS-R did not include its components as candidate variables. A missing indicator was used in modeling for unknown values for a category. A landmark analysis at day 100 post sample collection was used to compare patients who had received a transplant to those who did not. The Welch *t*-test was used to compare the average mutation rate between groups. All tests are reported as two-sided and considered significant at the <0.05 level. SAS version 9.4 and RStudio version 0.99.441 with R version 3.4.1 were used for all analyses.

## Results

### Complex karyotype MDS patients have a high frequency of TP53 mutations which are associated with specific clinical features

Of the 359 MDS patients with CK shared with the IWG for MDS Prognosis Molecular Committee, 339 (94%) had *TP53* sequencing performed. One or more mutations were identified in 186 (55%) cases. Patient characteristics stratified by *TP53* mutation status are shown in Table 1. Of the 186 *TP53* mutated patients, 164 (89%) were evaluable for multiple mutations and 159 (85%) could be analyzed for type of mutation.

**Table 1:** Patient demographics and laboratory values

	*N* (%)	*TP53* WT^a^	*TP53* mut^a^	*P* value^b^
*N*	359	153	186	
Age, median (range)	68 (23, 94)	67 (34, 89)	70 (23, 94)	0.096
<50 Years	28 (8)	12 (8)	15 (8)	0.22
50–59 Years	55 (15)	25 (16)	25 (13)	
60–69 Years	107 (30)	51 (33)	49 (26)	
70–80 Years	135 (37)	56 (37)	73 (39)	
≥80 Years	33 (10)	9 (6)	23 (12)	
Unknown	1 (<1)	0 (0)	1 (<1)	
Sex				
Male	223 (62)	102 (67)	107 (58)	0.093
Female	136 (38)	51 (33)	79 (42)	
Bone marrow blast %, median (range)	7 (0, 28)	5 (0, 27)	9 (0, 28)	<0.001
<5%	135 (38)	69 (45)	54 (29)	0.001
5–10%	104 (29)	39 (25)	59 (32)	
11–20%	101 (28)	35 (23)	65 (35)	
21–29%	6 (2)	2 (1)	3 (2)	
Unknown	13 (4)	8 (5)	5 (3)	
IPSS-R risk group				
Very low	0 (0)	0 (0)	0 (0)	<0.001
Low	5 (1)	4 (3)	1 (<1)	
Intermediate	26 (7)	15 (10)	6 (3)	
High	73 (20)	39 (25)	29 (16)	
Very high	224 (62)	78 (51)	136 (73)	
Unknown	31 (9)	17 (11)	14 (8)	
Hemoglobin, median (range)	9.4 (3.7, 17.0)	9.4 (3.7, 17.0)	9.2 (5.3, 13.5)	0.43
<8.0 (g/dL)	61 (17)	29 (19)	30 (16)	0.85
8.0–9.99 (g/dL)	161 (45)	67 (44)	85 (46)	
10.0–11.99 (g/dL)	102 (28)	40 (26)	55 (30)	
≥12.0 (g/dL)	23 (6)	14 (9)	7 (4)	
Unknown	12 (3)	3 (2)	9 (5)	
Absolute neutrophil count (ANC), median (range)	1.10 (0, 35.0)	1.31 (0, 17.27)	0.94 (0, 35.0)	0.22
<0.5 (×10^3^/μL)	62 (17)	28 (18)	32 (17)	0.49
0.5–1.8 (×10^3^/μL)	145 (40)	62 (41)	74 (40)	
1.8–9.99 (×10^3^/μL)	101 (28)	45 (29)	47 (25)	
≥10 (×10^3^/μL)	7 (2)	5 (3)	2 (1)	
Unknown	44 (12)	13 (8)	31 (17)	
Platelet count, median (range)	58 (4, 1073)	70 (5, 1073)	47 (5, 693)	0.002
<50 (×10^3^/μL)	152 (42)	50 (33)	93 (50)	<0.001
50–99 (×10^3^/μL)	89 (25)	40 (26)	46 (25)	
100–149 (×10^3^/μL)	49 (14)	24 (16)	22 (12)	
150–449 (×10^3^/μL)	46 (13)	26 (17)	15 (8)	
≥450 (×10^3^/μL)	5 (1)	4 (3)	1 (1)	
Unknown	18 (5)	9 (6)	9 (5)	

^a^*TP53* mutation status was unknown for 20 samples

^b^Test excludes unknown categories

As shown in Table 1, *TP53* mutations were associated with several prognostically adverse features. This included a lower median platelet count (47 vs. 70 × 10^9^/L, *p* = 0.002) and higher median bone marrow blast percentage (9% vs. 5%, *p* < 0.001), both of which are considered unfavorable risk factors in various prognostic scoring systems. No differences in hemoglobin level or absolute neutrophil counts were noted.

### TP53 mutations are associated with molecular and cytogenetic abnormalities

**Fig. 1:**
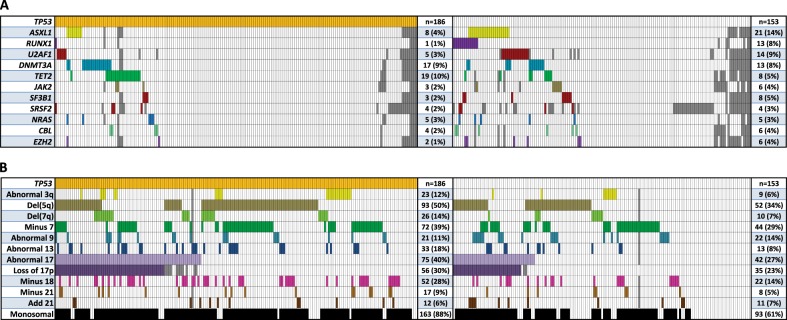
Select somatically mutated genes and karyotype abnormalities. **a** Co-mutation plot for somatically mutated genes in complex karyotype MDS patients with and without mutated *TP53* (left and right panels, respectively). Each column represents an individual patient. A colored bar indicates a mutation of the gene in that row with gray bars indicating missing data. The last column indicates the number of patients with a mutation of each gene. **b** Plot of recurrent karyotype abnormalities in patients with and without mutated *TP53* (left and right panels, respectively) using the same schema as in (**a**). *TP53* mutant patients had a higher rate of del(5q) abnormality (50% vs. 34%, *p* = 0.004), abnormal chromosome 13 (18% vs. 8%, *p* = 0.017), abnormal chromosome 17 (40% vs. 27%, *p* = 0.016), abnormal chromosome 18 (28% vs. 14%, *p* = 0.004), and del(7q) (14% vs. 7%, *p* = 0.033), but a lower rate of der(1;7)(q10;p10) ( < 1% vs. 5%, *p* = 0.025)

Complex karyotype MDS patients harbor fewer somatic point mutations in genes other than *TP53* when compared with non CK-MDS patients [13–15]. The majority of samples in our cohort were tested for somatic mutations in several recurrently mutated MDS genes (Supplemental Table 3). The most frequently mutated genes after *TP53* were *DNMT3A* (31/324, 10%), *ASXL1* (29/319, 9%), and *TET2* (27/318, 8%), all at rates lower than observed in MDS cohorts unselected by karyotype. Several gene mutations were even more underrepresented in the *TP53* mutant patient samples compared to wild-type CK-MDS (Fig. 1a, Supplemental Fig. 1). The *TP53* mutant group had fewer mutations of *ASXL1* (5% vs. 15%, *p* = 0.003), *U2AF1* (3% vs. 11%, p = 0.008), and *RUNX1* (0.5% vs. 9%, *p* < 0.001). A total of 250 patients had 12 core genes sequenced (*TP53*, *ASXL1*, *RUNX1*, *U2AF1*, *DNMT3A*, *TET2*, *JAK2*, *SF3B1*, *SRSF2*, *NRAS*, *CBL*, and *EZH2*). Of the 156 with mutated *TP53*, 111 (71%) had no additional gene mutations compared to 47 (50%) of the 94 without a *TP53* mutation (*p* = 0.001 by Fisher's exact test). The average number of mutated genes in the *TP53* mutant group was 0.39 non-*TP53* genes/patient, whereas in the *TP53* wild-type group, this ratio was 0.81 (*p* < 0.001 by Welch *t*-test).

*TP53* mutation status was also associated with the number and types of chromosomal abnormalities present within the complex karyotype. Del(5q), monosomy 7, and abnormalities of chromosome 17 were the most common recurrent cytogenetic findings, present in 156 (43%), 123 (34%), and 121 (34%) members of the entire cohort respectively (Fig. 1b, Supplemental Table 4).

**Fig. 2:**
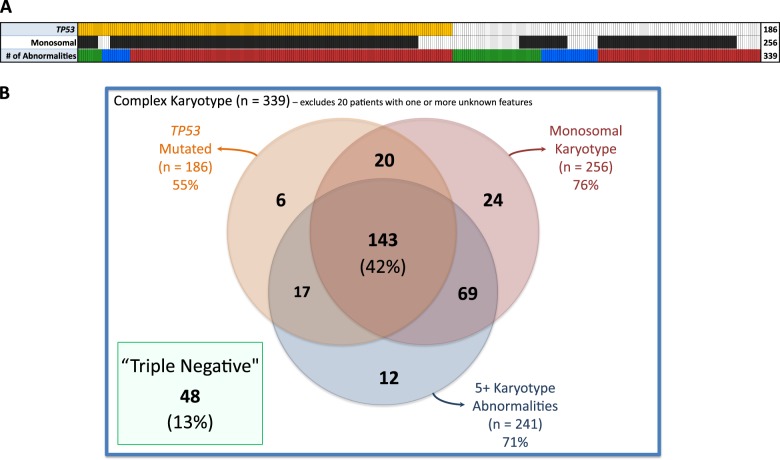
Interaction between *TP53* mutation, monosomy, and number of karyotype abnormalities. **a** Each column represents an individual patient with orange and black bars indicating *TP53* mutation and monosomal karyotype respectively. Colored bars in the last row indicate the number of karyotype abnormalities with green representing 3, blue representing 4, and red representing 5 or more. **b** Venn diagram showing number of cases with overlapping features

**Fig. 3:**
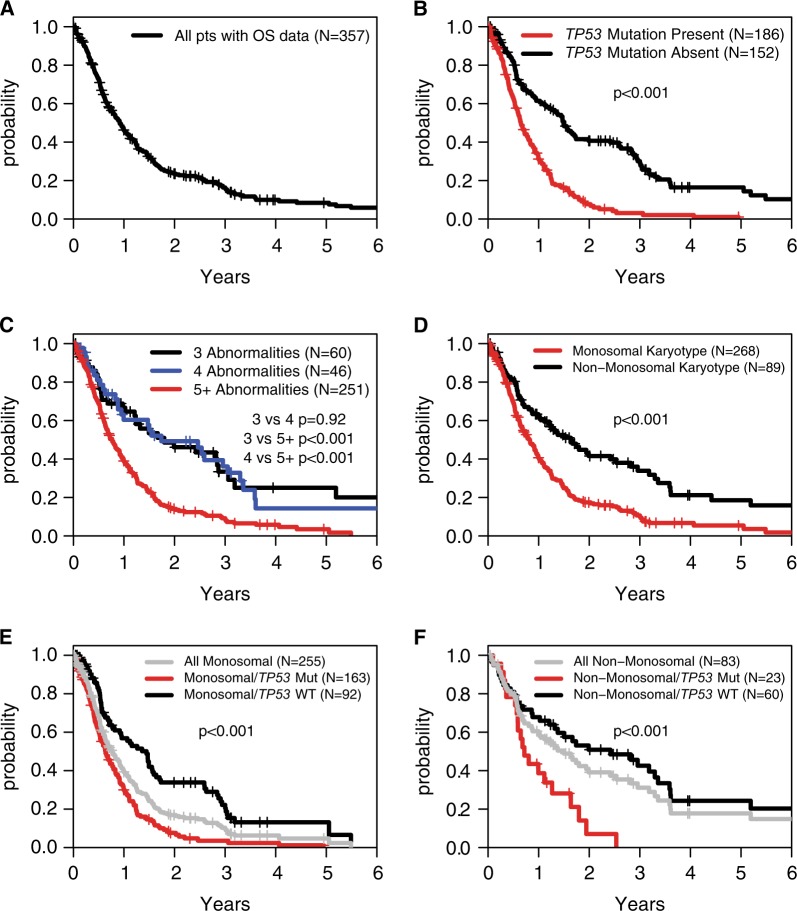
Overall survival by *TP53* mutation, high complexity, and monosomal karyotype status. **a** Overall survival of the entire cohort. **b** Overall survival stratified by *TP53* mutation status. **c** Overall survival stratified by the number of clonal karyotype abnormalities. **d** Overall survival stratified by monosomal karyotype status. **e** Stratification of overall survival by *TP53* mutation status in patients with a monosomal karyotype. **f** Stratification of overall survival by *TP53* mutation status in patients without a monosomal karyotype

Cases with five or more karyotype abnormalities were described as having ‘high complexity’ (HC) (Fig. 2a) given the marked difference in OS at this cut point (Fig. 3c). HC was found in 86% of *TP53* mutant patients compared with 53% of those without an identified *TP53* mutation (*p* < 0.001). *TP53* mutation status was also associated with MK, a feature that has frequently been cited as an independent prognostic measure in MDS and AML [7, 8, 11, 12, 31–34]. Eighty-eight percent of the *TP53* mutant patients had MK compared to 61% without the mutation (*p* < 0.001). These distinct methods of describing the complex karyotype, HC and MK, demonstrate significant overlap and association with *TP53* mutation status as 42% of patients harbored all three features (Fig. 2b).

### Karyotype abnormalities and TP53 mutation are associated with OS

As a group, this cohort with CK-MDS patients had a poor outcome, with a median OS of only 0.9 years (Fig. 3a). Even shorter OS might be expected in the *TP53* mutant subset given the associations between *TP53* mutation status and the adverse clinical and cytogenetic measures described above. Indeed, CK-MDS patients with *TP53* mutation had a significantly greater hazard ratio (HR) of death (2.57; 95% confidence interval (CI) 1.97–3.34, *p* < 0.001) with a median OS of 0.6 years compared to 1.5 years for *TP53* wild-type patients (Fig. 3b). No other gene mutation was significantly associated with OS in univariate analyses.

Prior studies of MDS patients unselected by karyotype have demonstrated that the prognostic significance of *TP53* mutations depends in part on their variant allele frequency (VAF), with smaller clones having a less adverse impact [27, 29]. To determine whether this holds true in complex karyotype MDS, we examined the survival of 151 patients with *TP53* mutations and available VAF data. Nearly two-thirds of *TP53* mutant patients had a VAF > 0.4, with a significantly shorter median OS than those with a VAF ≤ 0.4 (0.6 vs. 1.1 years, *p* = 0.004; Supplemental Fig. 2A). However, mutated patients with a *TP53* VAF ≤ 0.4 still had an inferior survival compared with *TP53* wild-type patients (1.1 vs. 1.5 years, *p* = 0.001). While *TP53* VAF was not adjusted for copy number in this analysis, the results were similar in the subset of patients without loss of 17p in their karyotype (*p* = 0.014 for *TP53* VAF ≤ 0.4 vs. > 0.40; Supplemental Fig. 2B).

The number and type of mutations in *TP53* had less impact on OS. Less than 15% of the cohort carried more than one *TP53* mutation, and this was not associated with any difference in survival compared to those harboring only 1 mutation (*p* = 0.77). In contrast, an increase in median OS was noted for missense mutations (*n* = 126) compared with potentially more disruptive types of mutations (frameshift, nonsense, and splice site; *n* = 33) among the 159 patients with mutation-type data available (*p* = 0.016; Supplemental Fig. 3). Complete loss of the *TP53* locus through deletion of chromosome 17p is not routinely captured by gene sequencing, but could have the same effect as a *TP53* mutation. However, cytogenetic abnormalities predicted to cause copy number loss at the *TP53* locus had no prognostic impact regardless of *TP53* mutations status (Supplemental Fig. 4), suggesting that loss of a *TP53* allele by cytogenetic analysis might not be biologically equivalent to a *TP53* point mutation in CK-MDS [2, 5, 35, 36]. Further testing of this hypothesis would require examination with more reliable methods including *TP53*-specific fluorescence in situ hybridization (FISH) probes or copy number-sensitive genomic arrays.

**Fig. 4:**
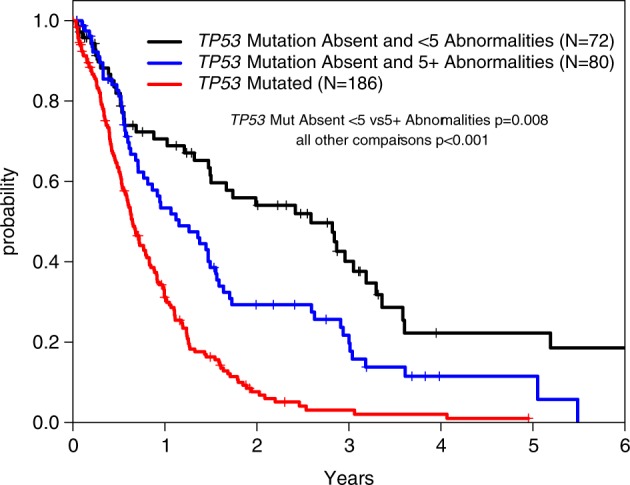
Overall survival stratified by *TP53* mutation and high complexity status

To determine how HC or MK could impact prognosis, we examined OS in patients stratified by these measures. Individually, MK and having five or more karyotype abnormalities were associated with inferior OS (Fig. 3c, d). However, in a multivariable model that considered *TP53* mutation, MK, and HC, the presence of MK was no longer statistically significant (Table 2). Indeed, *TP53* mutation status could strongly stratify survival of patients with and without MK (Fig. 3e, f). Double negative patients, defined as having neither *TP53* mutation nor HC, had markedly better outcomes with a median OS of 2.6 years compared with 0.6 years (*p* < 0.001) for *TP53* mutant and 1.2 years (*p* < 0.001) for *TP53* wild type but with HC (Fig. 4, Supplemental Figure 5).

**Table 2:** Overall survival modeling of *TP53* mutation and karyotype features

Overall survival model	Univariable		Multivariable	
Considered features	HR [95% CI]	*P* value	HR [95% CI]	*P* value
Monosomal yes vs. no	1.95 [1.46–2.62]	<0.001	1.26 [0.91–1.75]	0.17
Number of abnormalities ≥5 vs. 4 or 3	2.26 [1.70–3.02]	<0.001	1.61 [1.16–2.24]	0.004
*TP53* mutation vs. no mutation	2.57 [1.97–3.34]	<0.001	2.12 [1.61–2.79]	<0.001
Unknown vs. no mutation	0.70 [0.38–1.31]	0.27	0.69 [0.37–1.29]	0.25

### Multivariable prognostic modeling of OS

While the two-component model above can risk stratify CK-MDS patients, it does not consider the potential contributions of individual karyotype abnormalities, other gene mutations, or clinical measures that have significant univariate associations with OS (Supplemental Table 5). To explore the prognostic value of these features, we performed multivariable stepwise Cox regression modeling of OS in our cohort.

Candidate variables included age, sex, blood counts, bone marrow blast percentage, mutations in sequenced genes, and the presence of the specific karyotype abnormalities listed in Supplemental Table 5. *TP53* mutation was the most significant genetic risk factor, with a HR of 2.67 (Table 3) followed by mutations of *SF3B1* and *NRAS* (Supplemental Figure 6). Cytogenetic features in the final model included monosomy 7 and abnormalities of chromosomes 3q and 9. These factors had the greatest impact in patients without a *TP53* mutation, although monosomy 7 was associated with a shorter OS even in the *TP53* mutant group (Supplemental Figure 7). The only clinical factors to retain independent prognostic significance were elevated bone marrow blast percentage and low hemoglobin concentration (Supplemental Figure 8). Importantly, consideration of sample origin (univariate *p* = 0.18) during model building did not alter the significance of other covariates and was not retained (data not shown). Repeating the multivariable analysis with IPSS-R risk groups in place of bone marrow blast percentage and blood counts as candidate variables gave similar results with IPSS-R high (HR 3.27) and very high (HR 4.54) risk groups retained in the final model (Supplemental Table 6; Supplemental Figure 9). Most of the prior model variables remained significant with monosomy 21 as the only additional risk factor observed. In both models, *TP53* mutation status remained the most frequently occurring risk factor not currently considered by existing prognostic scoring systems.

**Table 3:** Cox regression modeling of overall survival

	Univariable HR [95% CI]	*P* value	Final multivariable HR [95% CI]	*P* value	*N* (%) non-reference group
**Final model** ^**a**^					
**Gene mutations**					
*TP53* mutation vs. no mutation	2.56 [1.96–3.33]	<0.001	2.67 [2.01–3.53]	<0.001	185 (52)
*Unknown vs. no mutation*	0.70 [0.38–1.31]	0.27	1.24 [0.46–3.36]	0.68	19 (5)
*SF3B1* mutation vs. no mutation^b^	1.26 [0.62–2.56]	0.52	2.81 [1.34–5.89]	0.006	11 (3)
*Unknown vs. no mutation*	1.24 [0.46–3.36]	0.68	0.75 [0.42–1.35]	0.34	33 (9)
*NRAS* mutation vs. no mutation^b^	1.79 [0.88–3.63]	0.11	2.50 [1.21–5.16]	0.013	10 (3)
*Unknown vs. no mutation*	0.61 [0.38–0.98]	0.043	0.89 [0.38–2.10]	0.79	33 (9)
**Cytogenetic abnormalities**					
−7 Yes vs. no	1.80 [1.40–2.31]	<0.001	1.66 [1.28–2.17]	<0.001	120 (34)
Abnormal 3q yes vs. no	1.99 [1.33–2.98]	<0.001	1.85 [1.23–2.79]	0.003	33 (9)
Abnormal 9 yes vs. no	1.47 [1.02–2.11]	0.037	1.90 [1.31–2.77]	<0.001	45 (13)
**Clinical features**					
Blast %					
5–10% vs. <5%	1.41 [1.03–1.91]	0.030	1.24 [0.90–1.71]	0.20	104 (29)
11–30% vs. <5%	2.05 [1.53–2.75]	<0.001	1.68 [1.24–2.29]	<0.001	106 (29)
*Unknown vs.* *<5%*	1.12 [0.60–2.09]	0.73	1.20 [0.63–2.30]	0.58	12 (3)
Hemoglobin (g/dL)					
10.0–11.99 vs. ≥12.0	1.97 [1.09–3.58]	0.025	1.30 [0.71–2.38]	0.40	102 (29)
8.0–9.99 vs. ≥12.0	2.71 [1.53–4.81]	<0.001	1.72 [0.96–3.11]	0.071	160 (45)
<8.0 vs. ≥12.0	3.67 [1.97–6.86]	<0.001	2.93 [1.53–5.62]	0.001	58 (16)
*Unknown vs.* *≥12.0*	2.60 [1.11–6.09]	0.028	1.52 [0.64–3.62]	0.35	12 (3)

^a^Modeling performed for 355 patients, excluding 2 patients with unknown survival status and 2 with incomplete karyotype information

^b^Of the 11 patients with *SF3B1* mutations, 3 also had *TP53* mutation, and of the 10 patients with *NRAS* mutations, 5 had a *TP53* mutation

## Discussion

Complex karyotype MDS includes a diverse collection of patients typically labeled as having a very poor prognosis [2, 4, 5]. Here we examined data from 359 CK-MDS patients evaluated at multiple centers around the world to determine which factors might improve current risk stratification methods. Collectively, these patients shared features that distinguished them from MDS patients without complex karyotypes. In addition to greater structural genomic instability and a high frequency of *TP53* mutations (55%), patients with CK-MDS had fewer somatic mutations in other MDS-associated genes. These differences were even more pronounced in the *TP53* mutant subset of CK-MDS, which were more likely to have high complexity, monosomal karyotypes, certain chromosomal abnormalities, and an even lower number of co-mutated myeloid malignancy genes. *TP53* mutant CK-MDS patients also had significantly higher bone marrow blast proportion and lower platelet counts, two factors strongly associated with elevated prognostic risk considered by clinical scoring systems like the IPSS-R. Indeed, *TP53* mutant CK-MDS patients had an OS that was less than half of that for non-mutant CK-MDS. This powerful adverse prognostic association was statistically independent of other risk factors, including having a higher number of karyotype abnormalities, which together overrode the prognostic impact of the monosomal karyotype.

The consideration of monosomal karyotype as a more accurate risk factor than karyotype complexity in MDS and AML has been controversial [9]. First, not all studies agree on the effect of MK on survival [6, 7, 10]. Second, the vast majority of studies examining the prognostic impact of MK in MDS did not evaluate *TP53* mutation status or HC, missing these potential confounders strongly associated with MK [8, 12, 37]. Finally, the definition of MK is not recognized by the International System for Human Cytogenetic Nomenclature (ISCN) and can be problematic to identify in practice [38, 39]. Some cases of apparent monosomies may be due to complicated unbalanced rearrangements and not truly representative of loss of a complete chromosome. Short of performing 24-color metaphase FISH, this can be difficult to measure reliably. Our results suggest that specific monosomies can retain prognostic significance after consideration of HC and *TP53* mutation status, but the more problematic designation of MK does not. Assessment of just HC and *TP53* mutation status constitutes a relatively simple means of identifying the roughly 20% of CK-MDS patients predicted to have an OS that resembles that of IPSS-R intermediate risk patients.

Consideration of multiple clinical, cytogenetic, and molecular features identifies *TP53* mutation among the most significant prognostic factor in patients with CK-MDS, yet it remains the only marker not routinely assessed in clinical practice. Here we demonstrate that the presence of *TP53* mutation has an independent impact on prognosis that is as great as having severe anemia and greater than having a bone marrow blast proportion of 10–29%. The muted impact of increased blast proportion and the absence of severe thrombocytopenia as independent risk factors are likely due to the association of these features with *TP53* mutations. The impact of a *TP53* mutation is pronounced even in patients assigned to the very high risk group by the IPSS-R (Supplemental Figure 9). Mutations of *SF3B1* and *NRAS*, while rare, were also associated with a greater HR of death. *NRAS* mutations are known to be adverse in a variety of contexts [40, 41], but *SF3B1* mutations are typically considered favorable in MDS [14, 15, 42, 43]. In the context of a complex karyotype, *SF3B1* mutations appear adverse, much like in rare cases of *SF3B1*-mutated AML [44]. Factors that might explain this association were not evident in our small subset of 11 *SF3B1* mutant cases. Future prognostic scoring systems that include molecular features will need to consider the interaction between somatic mutations and more traditional risk factors. In the meantime, patients with CK-MDS considered to have a poor prognosis with tools like the IPSS-R can be further risk stratified by consideration of the features in our survival model.

The value of identifying *TP53* mutations in MDS may extend beyond their prognostic significance. This study and others have demonstrated that *TP53* mutant MDS patients share clinical and genetic features that distinguish them from patients with wild-type *TP53*. In addition to a higher bone marrow blast proportion, lower platelet count, and greater likelihood of having a high number of chromosomal aberrations, *TP53* mutant patients relapse quickly after various forms of treatment [20, 22, 24, 45, 46]. Hematopoietic clones defined by *TP53* mutations are enriched after chemotherapy exposure and in therapy-related MDS, suggesting they harbor intrinsic resistance to genotoxic stress [47–50]. *TP53* mutations may also help select therapy. For example, novel agents, like APR-246 that specifically target missense mutations of *TP53* are in development [51]. As a consequence, *TP53* mutant CK-MDS could be considered a distinct subtype of disease with common genetic, clinical, and therapy-related features.

Potential limitations of this multi-center, retrospective analysis include possible differences in patient features and clinical practice patterns as well as the variety of sequencing methods and analysis pipelines at each institution. Not all centers reported the type, number, or VAFs of *TP53* mutations identified and data on time to AML transformation was not available. However, sample origin was not a significant confounder in our multivariable analyses. Information about treatment status was incomplete or absent in over a third of the cohort, although no disease-modifying therapy, including stem cell transplantation, has been definitively shown to mitigate the adverse impact of *TP53* mutation. Only 27 patients (8%) were reported as having received a stem cell transplant and the transplant status was not known for the majority of patients. Similarly, whether patients had primary vs. therapy-related MDS (t-MDS) was not known for 86 patients (24%). Only 21 patients (6%) were reported as having t-MDS. These measures had little impact on OS (Supplementary Figure 10).

## Conclusion

This study has several important strengths. It examines a large cohort of CK-MDS patients powered to find strong associations between clinical and genetic disease features including OS. It validates and expands upon results from many prior smaller studies. This consistency and the multi-institutional nature of the cohort imply that our conclusions are robust and generalizable. Finally, our findings support modifications to the standard of care for CK-MDS patients to include routine genetic sequencing of *TP53*. These mutations modify risk assessment even in CK-MDS patients traditionally considered to have the greatest disease risk. *TP53* mutation status is the most significant risk marker in this population missing from prognostic tools used in clinical practice. Cytogenetics alone appears insufficient for the evaluation of CK-MDS patients and routine testing for *TP53* mutations should be considered in this population.

## Supplementary information

Supplemental Tables and Figures

## References

[1] Bejar R (2014). Clinical and genetic predictors of prognosis in myelodysplastic syndromes. Haematologica.

[2] Schanz J, Tuchler H, Sole F (2012). New comprehensive cytogenetic scoring system for primary myelodysplastic syndromes (MDS) and oligoblastic acute myeloid leukemia after MDS derived from an international database merge. J Clin Oncol.

[3] Schanz J, Steidl C, Fonatsch C (2011). Coalesced multicentric analysis of 2,351 patients with myelodysplastic syndromes indicates an underestimation of poor-risk cytogenetics of myelodysplastic syndromes in the international prognostic scoring system. J Clin Oncol.

[4] Greenberg P, Cox C, LeBeau MM (1997). International scoring system for evaluating prognosis in myelodysplastic syndromes. Blood.

[5] Greenberg PL, Tuechler H, Schanz J (2012). Revised international prognostic scoring system for myelodysplastic syndromes. Blood.

[6] Anelli L, Pasciolla C, Zagaria A, Specchia G, Albano F (2017). Monosomal karyotype in myeloid neoplasias: a literature review. Onco Targets Ther.

[7] Patnaik MM, Hanson CA, Hodnefield JM, Knudson R, Van Dyke DL, Tefferi A (2011). Monosomal karyotype in myelodysplastic syndromes, with or without monosomy 7 or 5, is prognostically worse than an otherwise complex karyotype. Leukemia.

[8] Cluzeau T, Mounier N, Karsenti JM (2013). Monosomal karyotype improves IPSS-R stratification in MDS and AML patients treated with Azacitidine. Am J Hematol.

[9] Schanz J, Tuchler H, Sole F (2013). Monosomal karyotype in MDS: explaining the poor prognosis?. Leukemia.

[10] Valcarcel D, Adema V, Sole F (2013). Complex, not monosomal, karyotype is the cytogenetic marker of poorest prognosis in patients with primary myelodysplastic syndrome. J Clin Oncol.

[11] Yang YT, Hou HA, Liu CY (2014). IPSS-R in 555 Taiwanese patients with primary MDS: Integration of monosomal karyotype can better risk-stratify the patients. Am J Hematol.

[12] McQuilten ZK, Sundararajan V, Andrianopoulos N (2015). Monosomal karyotype predicts inferior survival independently of a complex karyotype in patients with myelodysplastic syndromes. Cancer.

[13] Bejar R, Stevenson K, Abdel-Wahab O (2011). Clinical effect of point mutations in myelodysplastic syndromes. N Engl J Med.

[14] Papaemmanuil E, Gerstung M, Malcovati L (2013). Clinical and biological implications of driver mutations in myelodysplastic syndromes. Blood.

[15] Haferlach T, Nagata Y, Grossmann V (2014). Landscape of genetic lesions in 944 patients with myelodysplastic syndromes. Leukemia.

[16] Haferlach C, Dicker F, Herholz H, Schnittger S, Kern W, Haferlach T (2008). Mutations of the TP53 gene in acute myeloid leukemia are strongly associated with a complex aberrant karyotype. Leukemia.

[17] Bally C, Ades L, Renneville A (2014). Prognostic value of TP53 gene mutations in myelodysplastic syndromes and acute myeloid leukemia treated with azacitidine. Leuk Res.

[18] Welch JS, Petti AA, Miller CA (2016). TP53 and decitabine in acute myeloid leukemia and myelodysplastic syndromes. N Engl J Med.

[19] Della Porta MG, Galli A, Bacigalupo A (2016). Clinical effects of driver somatic mutations on the outcomes of patients with myelodysplastic syndromes treated with allogeneic hematopoietic stem-cell transplantation. J Clin Oncol.

[20] Bejar R, Stevenson KE, Caughey B (2014). Somatic mutations predict poor outcome in patients with myelodysplastic syndrome after hematopoietic stem-cell transplantation. J Clin Oncol.

[21] Lindsley RC, Saber W, Mar BG (2017). Prognostic mutations in myelodysplastic syndrome after stem-cell transplantation. N Engl J Med.

[22] Montalban-Bravo G, Takahashi K, Patel K (2018). Impact of the number of mutations in survival and response outcomes to hypomethylating agents in patients with myelodysplastic syndromes or myelodysplastic/myeloproliferative neoplasms. Oncotarget.

[23] Craddock CF, Houlton AE, Quek LS (2017). Outcome of azacitidine therapy in acute myeloid leukemia is not improved by concurrent vorinostat therapy but is predicted by a diagnostic molecular signature. Clin Cancer Res.

[24] Bejar R, Lord A, Stevenson K (2014). TET2 mutations predict response to hypomethylating agents in myelodysplastic syndrome patients. Blood.

[25] Yoshizato T, Nannya Y, Atsuta Y (2017). Genetic abnormalities in myelodysplasia and secondary acute myeloid leukemia: impact on outcome of stem cell transplantation. Blood.

[26] Kulasekararaj AG, Smith AE, Mian SA (2013). TP53 mutations in myelodysplastic syndrome are strongly correlated with aberrations of chromosome 5, and correlate with adverse prognosis. Br J Haematol.

[27] Sallman DA, Komrokji R, Vaupel C (2016). Impact of TP53 mutation variant allele frequency on phenotype and outcomes in myelodysplastic syndromes. Leukemia.

[28] Goel S, Hall J, Pradhan K (2016). High prevalence and allele burden-independent prognostic importance of p53 mutations in an inner-city MDS/AML cohort. Leukemia.

[29] Wang W, Routbort MJ, Tang Z (2017). Characterization of TP53 mutations in low-grade myelodysplastic syndromes and myelodysplastic syndromes with a non-complex karyotype. Eur J Haematol.

[30] Malcovati L, Galli A, Travaglino E (2017). Clinical significance of somatic mutation in unexplained blood cytopenia. Blood.

[31] Breems DA, Van Putten WL, De Greef GE (2008). Monosomal karyotype in acute myeloid leukemia: a better indicator of poor prognosis than a complex karyotype. J Clin Oncol.

[32] Kayser S, Zucknick M, Dohner K (2012). Monosomal karyotype in adult acute myeloid leukemia: prognostic impact and outcome after different treatment strategies. Blood.

[33] Koenecke C, Gohring G, de Wreede LC (2015). Impact of the revised International Prognostic Scoring System, cytogenetics and monosomal karyotype on outcome after allogeneic stem cell transplantation for myelodysplastic syndromes and secondary acute myeloid leukemia evolving from myelodysplastic syndromes: a retrospective multicenter study of the European Society of Blood and Marrow Transplantation. Haematologica.

[34] Wudhikarn K, Van Rheeden R, Leopold C, Rattanaumpawan P, Gingrich R, de Magalhaes Silverman M (2012). Outcome of allogeneic stem cell transplantation in myelodysplastic syndrome patients: prognostic implication of monosomal karyotype. Eur J Haematol.

[35] Stengel A, Kern W, Haferlach T, Meggendorfer M, Fasan A, Haferlach C (2017). The impact of TP53 mutations and TP53 deletions on survival varies between AML, ALL, MDS and CLL: an analysis of 3307 cases. Leukemia.

[36] Sebaa A, Ades L, Baran-Marzack F (2012). Incidence of 17p deletions and TP53 mutation in myelodysplastic syndrome and acute myeloid leukemia with 5q deletion. Genes Chromosomes Cancer.

[37] Itzykson R, Thepot S, Eclache V (2011). Prognostic significance of monosomal karyotype in higher risk myelodysplastic syndrome treated with azacitidine. Leukemia.

[38] McGowan-Jordan J, Simons A, Schmid M (eds). An International System for Human Cytogenomic Nomenclature (2016). Cytogenetic and Genome Research. Basel, Switzerland 2016;149. 10.1159/000516655 34407535

[39] Galvan AB, Mallo M, Arenillas L (2010). Does monosomy 5 really exist in myelodysplastic syndromes and acute myeloid leukemia?. Leuk Res.

[40] Murphy DM, Bejar R, Stevenson K (2013). NRAS mutations with low allele burden have independent prognostic significance for patients with lower risk myelodysplastic syndromes. Leukemia.

[41] Takahashi K, Jabbour E, Wang X (2013). Dynamic acquisition of FLT3 or RAS alterations drive a subset of patients with lower risk MDS to secondary AML. Leukemia.

[42] Malcovati L, Karimi M, Papaemmanuil E (2015). SF3B1 mutation identifies a distinct subset of myelodysplastic syndrome with ring sideroblasts. Blood.

[43] Malcovati L, Papaemmanuil E, Ambaglio I (2014). Driver somatic mutations identify distinct disease entities within myeloid neoplasms with myelodysplasia. Blood.

[44] Papaemmanuil E, Gerstung M, Bullinger L (2016). Genomic classification and prognosis in acute myeloid leukemia. N Engl J Med.

[45] Jadersten M, Saft L, Smith A (2011). TP53 mutations in low-risk myelodysplastic syndromes with del(5q) predict disease progression. J Clin Oncol.

[46] Scharenberg C, Giai V, Pellagatti A (2017). Progression in patients with low- and intermediate-1-risk del(5q) myelodysplastic syndromes is predicted by a limited subset of mutations. Haematologica.

[47] Lindsley RC, Mar BG, Mazzola E (2015). Acute myeloid leukemia ontogeny is defined by distinct somatic mutations. Blood.

[48] Wong TN, Miller CA, Jotte MRM (2018). Cellular stressors contribute to the expansion of hematopoietic clones of varying leukemic potential. Nat Commun.

[49] Gibson CJ, Lindsley RC, Tchekmedyian V (2016). Clonal hematopoiesis associated with adverse outcomes following autologous stem cell transplantation for non-Hodgkin lymphoma. Blood.

[50] Coombs CC, Zehir A, Devlin SM (2017). Therapy-related clonal hematopoiesis in patients with non-hematologic cancers is common and associated with adverse clinical outcomes. Cell Stem Cell.

[51] Deneberg S, Cherif H, Lazarevic V (2016). An open-label phase I dose-finding study of APR-246 in hematological malignancies. Blood Cancer J.

