# Supplementary material for: TP53 mutation status divides myelodysplastic syndromes with complex karyotypes into distinct prognostic subgroups
Source: Leukemia. 2019 Jan 11;33(7):1747–58. doi: 10.1038/s41375-018-0351-2 (PMC6609480; doi:10.1038/s41375-018-0351-2)

**Supplemental Tables and Figures**

Supplemental Table 1 – International Centers Contributing Study Samples

Supplemental Table 2 – How karyotype parsing was performed

Supplemental Table 3 – List of Somatic Mutations in Select Genes

Supplemental Table 4 – Cytogenetics by *TP53* mutational status

Supplemental Table 5 – Univariate Cox Regression Modeling

Supplemental Table 6 – Multivariate Cox Regression Modeling with IPSS-R Risk Groups

Supplemental Figure 1 – Mutationgram with parsed cytogenetic features

Supplemental Figure 2 – Survival by *TP53* mutation VAF

Supplemental Figure 3 – Survival by number and type of *TP53* mutation

Supplemental Figure 4 – Survival by 17p loss and *TP53* mutation

Supplemental Figure 5 – Survival by number of karyotype abnormalities and *TP53* mutation

Supplemental Figure 6 – Survival of *SF3B1* and *NRAS* mutated patients by *TP53* mutation status

Supplemental Figure 7 – Survival of abnl3, abnl9, and -7 patients by *TP53* mutation status

Supplemental Figure 8 – Survival by clinical parameters

Supplemental Figure 9 – Survival by IPSS-R risk group and *TP53* mutation

Supplemental Figure 10 – Survival by tMDS, treatment, and HSCT status

**Supplemental Table 1: International Centers Contributing Study Samples**

| Country | Contributing Center |
| --- | --- |
| Canada | University of British Columbia |
| France | Hopital Cochin, Assitance Publique-Hopitaux De Paris |
| France | Hôpital St Louis, Assistance Publique-Hôpitaux de Paris |
| Germany | Hannover Medical School |
| Germany | MLL Munich Leukemia Laboratory |
| Germany | University Medical Center, Georg-August University |
| Italy | Fondazione IRCCS Policlinico San Matteo & University of Pavia |
| Italy | University of Florence Medical School |
| Sweden | Karolinska Institutet, Karolinska University Hospital |
| Taiwan | Chang Gung Memorial Hospital and Chang Gung University |
| UK | MRC Molecular Hematology Unit, WIMM, University of Oxford, UK |
| UK | Radcliffe Department of Medicine, University of Oxford |
| UK | St. James's Institute of Oncology, Leeds Teaching Hospitals |
| UK | University of Dundee, Ninewells Hospital |
| USA | Cleveland Clinic Taussig Cancer Center |
| USA | Dana-Farber Cancer Institute |
| USA | H. Lee Moffitt Cancer Center and Research Institute |
| USA | University of Texas MD Anderson Cancer Center |
| USA | Vanderbilt-Ingram Cancer Center |
| USA | Washington University School of Medicine |

**Supplemental Table 2: Approach to parsing complex karyotypes with examples**

General Rules (adapted and revised from Chun *et al***†**):

- Non-clonal abnormalities present in only 1 metaphase are ignored.
- Count 1 aberration for each item between commas in the ISCN karyotype nomenclature string.
- If there are several clones, the one with the highest number of aberrations is counted independently whether it is a dependent subclone or an independent clone. (see Example 4).
- Count only 1 aberration for each numerical change (including –Y), balanced translocation, and simple structural change.
- If there is an unbalanced translocation this is counted as 2 aberrations – the initial translocation and then the loss of a derivative chromosome (see Example 7).
- Do not count constitutional aberrations, but if in doubt, count as 1 aberration.
- Only count 1 aberration for tetraploidy.
- Abnormalities in independent clones are not added together (see Example 1).
- For this study, a chromosome was considered abnormal if it was absent (monosomy), duplicated, translocated, inverted, iso- or dicentric, ring, or partially deleted.

| **Ex #** | **ISCN-Formula** | **Number** | **Explanatory Remarks** |
| --- | --- | --- | --- |
| **1** | 47,XX,+8,+21[11]/46,XX,del(5)(q13q33), ‑7[5] | 2 | Two independent clones, each with two abnormalties. This is not complex. |
| **2** | 45,XY,‑7,del(12)(p12),del(20)(q11.2q13.1)[20] | 3 |  |
| **3** | 45,XY,del(5)(q13q33),‑7,add(17)(q25), del(20)(q11.2)[7]/46,XY[13] | 4 |  |
| **4** | 48,XX,+8,+19,del(20)(q11.2)[4]/49,idem,+del(11)(?q23q23)[10]/46,XY[6] | 4 | Two dependent clones. The stem clone contains 3 changes, the subclone contains 4 aberrations and is counted |
| **5** | 46,XY,del(20)(q11.2)[9]/46,XY,add(7)(q11.2),‑13,‑17,i(21)(q10),+2mar[cp2]/46,XY[9] | >4 | 2 independent clones. The clone with the highest numer of aberrations was counted |
| **6** | 43~47,Y,‑X,add(1)(p34),add(2)(p11.2),‑4,+6,i(6)(p10),‑7,‑9,add(11)(p15),‑13,‑14,‑15,‑18,‑19,‑20,‑21,+r,+5~10mar[cp8]/46,XY[12] | >4 | Highly complex aberrant clone with cell-to-cell variations |
| **7** | 45,XY,del(5)(q12q34),der(8)t(1;8)(p31;p22), der(17;21) (q10;q10) | >4 | Besides the 5q-deletion, two unbalanced translocations leading to partial trisomy of 1p, partial monosomy of 8p, partial monosomy of 17p and partial monosomy of 21q are present. This adds up to 5 aberrations. According to Chun et al. these cases would have been considered as having only 3 aberrations. |

**†**Chun K, Hagemeijer A, Iqbal A, Slovak ML. *Leukemia Research*. 2010 Feb;34(2):160-5.

**Supplemental Table 3: Somatic Mutations by *TP53* mutational status**

|  | N (%) Sequenced (%) | N (%) Unknown | Number Mutated (%) | *TP53* Unmutated (%) | *TP53*  Mutated (%) | p-value† |
| --- | --- | --- | --- | --- | --- | --- |
| N | 359 | 359 |  | 153 | 186 |  |
| ***TP53*** | 339 (94) | 20 (6) | 186 (55) |  |  |  |
| ***DNMT3A*** | 324 (90) | 35 (10) | 31 (10) | 13 (9) | 17 (10) | >0.99 |
| ***ASXL1*** | 319 (89) | 40 (11) | 29 (9) | 21 (15) | 8 (5) | 0.003 |
| ***TET2*** | 318 (89) | 41 (11) | 27 (8) | 8 (6) | 19 (11) | 0.16 |
| ***U2AF1*** | 315 (88) | 44 (12) | 21 (7) | 14 (11) | 5 (3) | 0.008 |
| ***RUNX1*** | 329 (92) | 30 (8) | 15 (5) | 13 (9) | 1 (<1) | <0.001 |
| ***JAK2*** | 322 (90) | 37 (10) | 10 (3) | 6 (4) | 3 (2) | 0.19 |
| ***SF3B1*** | 326 (91) | 33 (9) | 11 (3) | 8 (6) | 3 (2) | 0.067 |
| ***CBL*** | 319 (89) | 40 (11) | 10 (3) | 6 (4) | 4 (2) | 0.34 |
| ***NRAS*** | 325 (91) | 34 (9) | 10 (3) | 5 (3) | 5 (3) | 0.76 |
| ***EZH2*** | 311 (87) | 48 (13) | 8 (3) | 6 (4) | 2 (1) | 0.082 |
| ***SRSF2*** | 282 (79) | 77 (21) | 8 (3) | 4 (4) | 4 (2) | 0.72 |
| *KRAS* | 246 (69) | 113 (31) | 7 (3) | 3 (3) | 4 (3) | 0.99 |
| *IDH1* | 332 (92) | 27 (8) | 2 (<1) | 1 (<1) | 1 (<1) | >0.99 |
| *IDH2* | 334 (93) | 25 (7) | 3 (<1) | 1 (<1) | 2 (1) | >0.99 |

† Fisher exact unknown categories excluded from test

The 12 genes in **bold** represent those sequenced in a core set of 250 patients

**Supplemental Table 4: Cytogenetics by *TP53* mutational status**

|  | N (%) | *TP53* WT | *TP53* mut | p-value† |
| --- | --- | --- | --- | --- |
| Total | 359 | 153 | 186 |  |
| Monosomal |  |  |  |  |
| Yes | 270 (75) | 93 (61) | 163 (88) | <0.001 |
| No | 89 (25) | 60 (39) | 23 (12) |  |
| Abnormal 17 |  |  |  |  |
| Yes | 121 (34) | 42 (27) | 75 (40) | 0.016 |
| No | 237 (66) | 110 (72) | 111 (60) |  |
| Unknown | 1 (<1) | 1 (<1) | 0 (0) |  |
| 17p loss of TP53 |  |  |  |  |
| Yes | 95 (26) | 35 (23) | 56 (32) | 0.084 |
| No | 251 (70) | 116 (76) | 119 (64) |  |
| Unknown | 13 (4) | 2 (1) | 11 (6) |  |
| -7 |  |  |  |  |
| Yes | 123 (34) | 44 (29) | 72 (39) | 0.052 |
| No | 235 (65) | 109 (71) | 113 (61) |  |
| Unknown | 1 (<1) | 0 (0) | 1 (<1) |  |
| del(7q) |  |  |  |  |
| Yes | 38 (11) | 10 (7) | 26 (14) | 0.033 |
| No | 319 (89) | 142 (93) | 159 (85) |  |
| Unknown | 2 (<1) | 1 (<1) | 1 (<1) |  |
| del(5q) |  |  |  |  |
| Yes | 156 (43) | 52 (34) | 93 (50) | 0.004 |
| No | 201 (56) | 100 (65) | 92 (50) |  |
| Unknown | 2 (<1) | 1 (<1) | 1 (<1) |  |
| Abnormal 3q |  |  |  |  |
| Yes | 33 (9) | 9 (6) | 23 (12) | 0.060 |
| No | 324 (90) | 143 (93) | 162 (87) |  |
| Unknown | 2 (<1) | 1 (<1) | 1 (<1) |  |
| der(1;7) |  |  |  |  |
| Yes | 9 (3) | 7 (5) | 1 (<1) | 0.025 |
| No | 348 (97) | 145 (95) | 184 (99) |  |
| Unknown | 2 (<1) | 1 (<1) | 1 (<1) |  |
| Abnormal 9 |  |  |  |  |
| Yes | 45 (13) | 22 (14) | 21 (11) | 0.42 |
| No | 312 (87) | 130 (85) | 164 (88) |  |
| Unknown | 2 (<1) | 1 (<1) | 1 (<1) |  |
| -13/13q |  |  |  |  |
| Yes | 48 (13) | 13 (8) | 33 (18) | 0.017 |
| No | 310 (86) | 139 (91) | 153 (82) |  |
| Unknown | 1 (<1) | 1 (<1) | 0 (0) |  |
| -18/18q |  |  |  |  |
| Yes | 81 (23) | 22 (14) | 52 (28) | 0.004 |
| No | 277 (77) | 130 (85) | 134 (72) |  |
| Unknown | 1 (<1) | 1 (<1) | 0 (0) |  |
| -21 |  |  |  |  |
| Yes | 26 (7) | 8 (5) | 17 (9) | 0.21 |
| No | 331 (92) | 144 (94) | 168 (90) |  |
| Unknown | 2 (<1) | 1 (<1) | 1 (<1) |  |
| +21 |  |  |  |  |
| Yes | 25 (7) | 11 (7) | 12 (6) | 0.83 |
| No | 332 (92) | 141 (92) | 173 (93) |  |
| Unknown | 2 (<1) | 1 (<1) | 1 (<1) |  |

†Test excludes unknown categories.

**Supplemental Table 5: Univariate Cox Regression Modeling**

|  | Univariable |  |
| --- | --- | --- |
|  | HR [95% CI] | p-value |
| Clinical Features |  |  |
| Age, >=70 vs. < 70 yrs.† | 1.37 [1.08-1.74] | 0.010 |
| Sex, M vs. F | 0.95 [0.74-1.22] | 0.68 |
| IPSS-R |  |  |
| High vs. Intermediate/Low | 3.49 [1.77-6.88] | <0.001 |
| Very High vs. Intermediate/Low | 5.57 [2.93-10.56] | <0.001 |
| Unknown vs. Intermediate/Low | 3.99 [1.92-8.27] | <0.001 |
| BM Blast % |  |  |
| 5-10% vs. < 5% | 1.41 [1.04-1.91] | 0.029 |
| 11-30% vs. < 5% | 2.08 [1.55-2.80] | <0.001 |
| Unknown vs. < 5% | 1.79 [0.78-4.11] | 0.73 |
| Hemoglobin (g/dL) |  |  |
| 10.0 – 11.99 vs. ≥12.0 | 1.97 [1.09-3.58] | 0.025 |
| 8.0 – 9.99 vs. ≥12.0 | 2.72 [1.53-4.82] | <0.001 |
| < 8.0 vs. ≥12.0 | 3.64 [1.96-6.77] | <0.001 |
| Unknown vs. ≥12.0 | 2.60 [1.11-6.10] | 0.028 |
| Absolute Neutrophil Count (x10^3^/μl) |  |  |
| 0.5 – 1.8 vs. < 0.5 | 0.79 [0.57-1.10] | 0.16 |
| 1.8 – 9.99 vs. <0.5 | 0.65 [0.45-0.93] | 0.018 |
| ≥10 vs. < 0.5 | 0.41 [0.10-1.67] | 0.21 |
| Unknown vs. < 0.5 | 1.04 [0.69-1.59] | 0.84 |
| Platelet Count (x10^3^/μl) |  |  |
| 50 – 99 vs. < 50 | 0.83 [0.61-1.11] | 0.20 |
| 100 – 149 vs. <50 | 0.50 [0.34-0.73] | <0.001 |
| 150 – 449 vs. <50 | 0.56 [0.38-0.83] | 0.004 |
| ≥450 vs. < 50 | 1.07 [0.34-3.38] | 0.90 |
| Unknown vs. < 50 | 0.87 [0.51-1.49] | 0.61 |
| Cytogenetic Abnormalities* |  |  |
| Monosomal Yes vs. No | 1.95 [1.45-2.62] | <0.001 |
| Number Abnormalities ≥5 vs. 3 or 4 | 2.26 [1.69-3.02] | <0.001 |
| Abnormal 17 Yes vs. No | 1.41 [1.09-1.81] | 0.008 |
| 17p loss of TP53 Yes vs. No | 1.41 [1.07-1.85] | 0.015 |
| -7 Yes vs. No | 1.80 [1.40-2.31] | <0.001 |
| del(7q) Yes vs. No | 1.20 [0.83-1.74] | 0.33 |
| del(5q) Yes vs. No | 1.24 [0.98-1.58] | 0.076 |
| Abnormal 3q Yes vs. No | 2.00 [1.33-2.98] | <0.001 |
| der(1;7) Yes vs. No | 1.00 [0.49-2.02] | 0.99 |
| Abnormal 9 Yes vs. No | 1.47 [1.02-2.11] | 0.037 |
| -13/13q Yes vs. No | 1.64 [1.16-2.31] | 0.005 |
| -18/18q Yes vs. No | 1.46 [1.10-1.93] | 0.009 |
| -21 Yes vs. No | 1.51 [0.98-2.35] | 0.064 |
| +21 Yes vs. No | 0.83 [0.53-1.30] | 0.43 |
| Gene Mutations (all vs. no mutation) |  |  |
| *TP53* Mutation | 2.57 [1.97-3.34] | <0.001 |
| *TP53* Unknown | 0.70 [0.38-1.31] | 0.27 |
| *ASXL1* Mutation | 0.72 [0.47-1.10] | 0.13 |
| *ASXL1* Unknown | 0.95 [0.64-1.40] | 0.78 |
| *DNMT3A* Mutation | 1.18 [0.78-1.77] | 0.43 |
| *DNMT3A* Unknown | 0.87 [0.57-1.34] | 0.53 |
| *TET2* Mutation | 1.12 [0.73-1.72] | 0.62 |
| *TET2* Unknown | 1.04 [0.71-1.51] | 0.85 |
| *U2AF1* Mutation | 0.85 [0.51-1.41] | 0.53 |
| *U2AF1* Unknown | 0.80 [0.54-1.19] | 0.27 |
| *RUNX1* Mutation | 0.77 [0.41-1.46] | 0.43 |
| *RUNX1* Unknown | 0.60 [0.36-0.98] | 0.040 |
| *JAK2* Mutation | 0.45 [0.19-1.09] | 0.078 |
| *JAK2* Unknown | 0.75 [0.49-1.15] | 0.19 |
| *SF3B1* Mutation | 1.26 [0.62-2.55] | 0.52 |
| *SF3B1* Unknown | 0.69 [0.44-1.07] | 0.10 |
| *CBL* Mutation | 1.02 [0.51-2.07] | 0.95 |
| *CBL* Unknown | 0.75 [0.50-1.12] | 0.16 |
| *NRAS* Mutation | 1.79 [0.88-3.62] | 0.11 |
| *NRAS* Unknown | 0.61 [0.38-0.98] | 0.043 |
| *KRAS* Mutation | 0.65 [0.27-1.59] | 0.34 |
| *KRAS* Unknown | 1.02 [0.79-1.32] | 0.88 |
| *EZH2* Mutation | 1.15 [0.43-3.10] | 0.78 |
| *EZH2* Unknown | 0.94 [0.66-1.34] | 0.74 |
| *SRSF2* Mutation | 1.09 [0.48-2.46] | 0.83 |
| *SRSF2* Unknown | 0.80 [0.59-1.07] | 0.13 |

†Excludes 1 patient with unknown age.

*Modeling performed for 355 patients, excluding 2 patients with unknown survival status and 2 with unknown cytogenetics.

**Supplemental Table 6: Multivariate Cox Regression Modeling with IPSS-R Risk Groups**

| **Model 2*†** |  |  |  |  |  |
| --- | --- | --- | --- | --- | --- |
| **IPSS-R Risk Groups** |  |  |  |  |  |
| Very High vs. Intermediate/Low | 5.56 [2.93-10.55] | <0.001 | 4.54 [2.33-8.83] | <0.001 | 221 (62) |
| High vs. Intermediate/Low | 3.48 [1.76-6.87] | <0.001 | 3.27 [1.62-6.59] | <0.001 | 73 (21) |
| *Unknown vs. Intermediate/Low* | 3.98 [1.92-8.26] | <0.001 | 3.54 [1.66-7.57] | 0.001 | *30 (8)* |
| **Gene Mutations** |  |  |  |  |  |
| *TP53* mutation vs. No mutation | 2.56 [1.96-3.33] | <0.001 | 2.38 [1.81-3.15] | <0.001 | 185 (52) |
| *Unknown vs. No mutation* | *0.70 [0.38-1.31]* | *0.27* | *1.24 [0.45-3.41]* | *0.67* | *19 (5)* |
| *SF3B1* mutation vs. No mutation** | 1.26 [0.62-2.56] | 0.52 | 3.01 [1.45-6.24] | 0.003 | 11 (3) |
| *Unknown vs. No mutation* | *1.24 [0.46-3.36]* | *0.68* | *1.04 [0.60-1.83]* | *0.88* | 33 (9) |
| *NRAS* mutation vs. No mutation | 1.79 [0.88-3.63] | 0.11 | 2.90 [1.40-5.99] | 0.004 | 10 (3) |
| *Unknown vs. No mutation* | *0.61 [0.38-0.98]* | *0.043* | *0.79 [0.33-1.87]* | *0.59* | *33 (9)* |
| **Cytogenetic Abnormalities** |  |  |  |  |  |
| -7 Yes vs. No | 1.80 [1.40-2.31] | <0.001 | 1.63 [1.26-2.11] | <0.001 | 120 (34) |
| Abnormal 3q Yes vs. No | 1.99 [1.33-2.98] | <0.001 | 1.80 [1.19-2.71] | 0.005 | 33 (9) |
| Abnormal 9 Yes vs. No | 1.47 [1.02-2.11] | 0.037 | 2.05 [1.41-2.98] | <0.001 | 45 (13) |
| -21 Yes vs. No | 1.52 [0.98-2.35] | 0.064 | 1.70 [1.07-2.71] | 0.025 | 26 (7) |

*Modeling performed for 355 patients, excluding 2 patients with unknown survival status and 2 with incomplete karyotype information. †Does not consider the clinical components of IPSS-R classification and due to the small numbers in the low category (n=5), low and intermediate categories were combined for modeling as the reference group. **Of the 11 patients with *SF3B1* mutations, 3 also had *TP53* mutation and of the 10 patients with *NRAS* mutations 5 had a *TP53* mutation.

**Supplemental Figure 1.** Co-mutation and Cytogenetic Abnormalities Figure for 359 Complex Karyotype MDS Patients


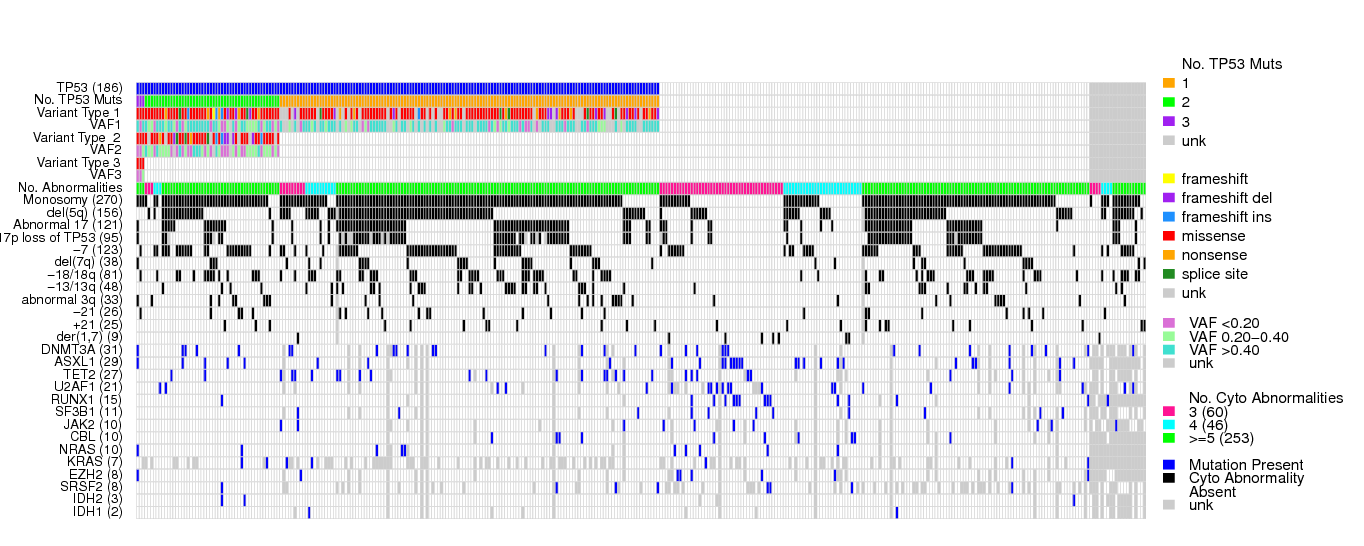


**Supplemental Figure 2:** Overall survival by *TP53* VAF in A) all patients with VAF information available and B) in the subset of these that did not have karyotype evidence of 17p deletion.


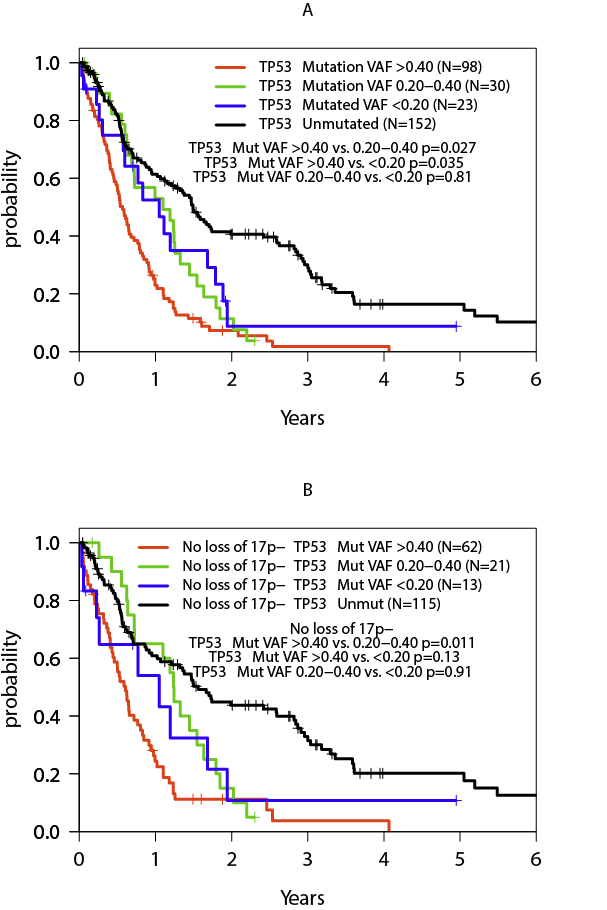


**Supplemental Figure 3:** Overall survival by A) type and B) number of *TP53* mutations in the subsets of patients with the relevant mutation data available


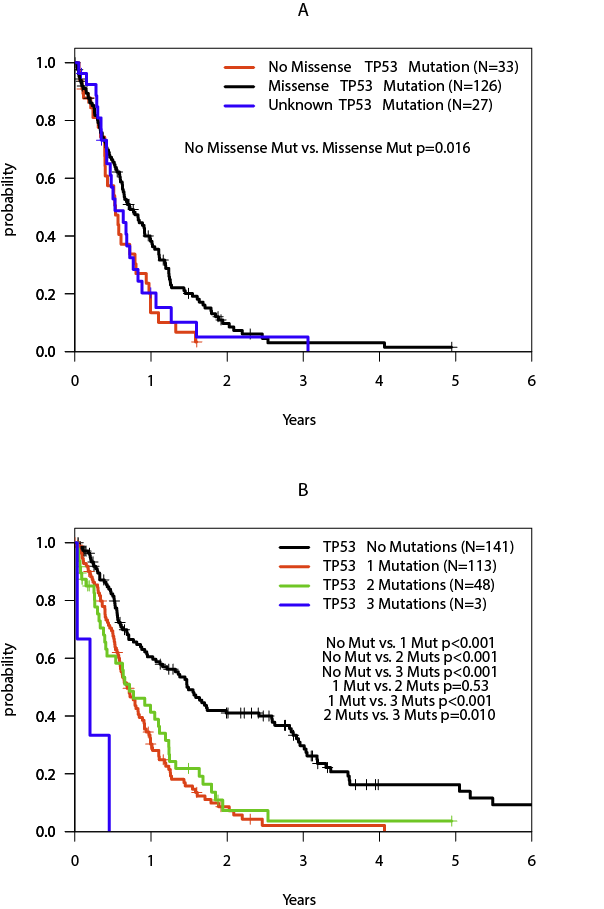


**Supplemental Figure 4:** Survival curves comparing the impact of del(17p) with predicted loss of the *TP53* locus in patients with and without *TP53* mutation.


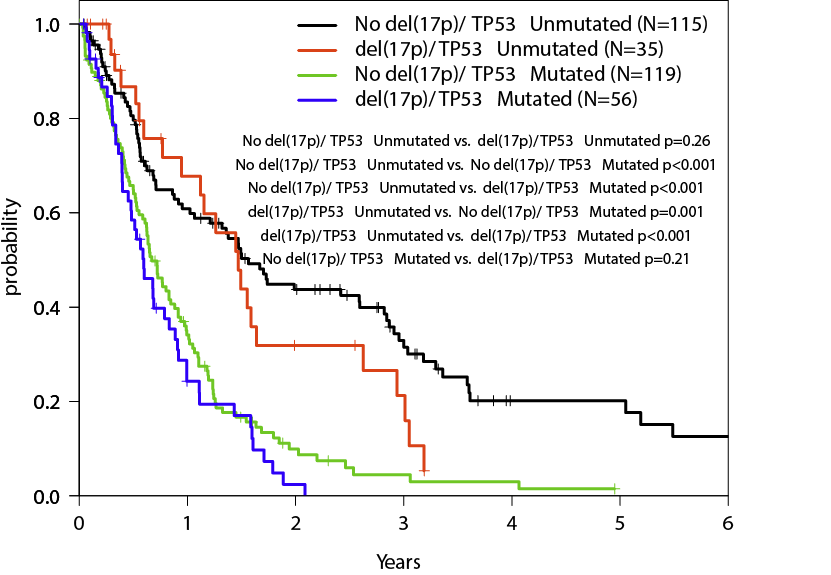


**Supplemental Figure 5:** Overall survival in patients with 3, 4, and 5 or more (HC) karyotype abnormalities stratified by *TP53* mutation status.


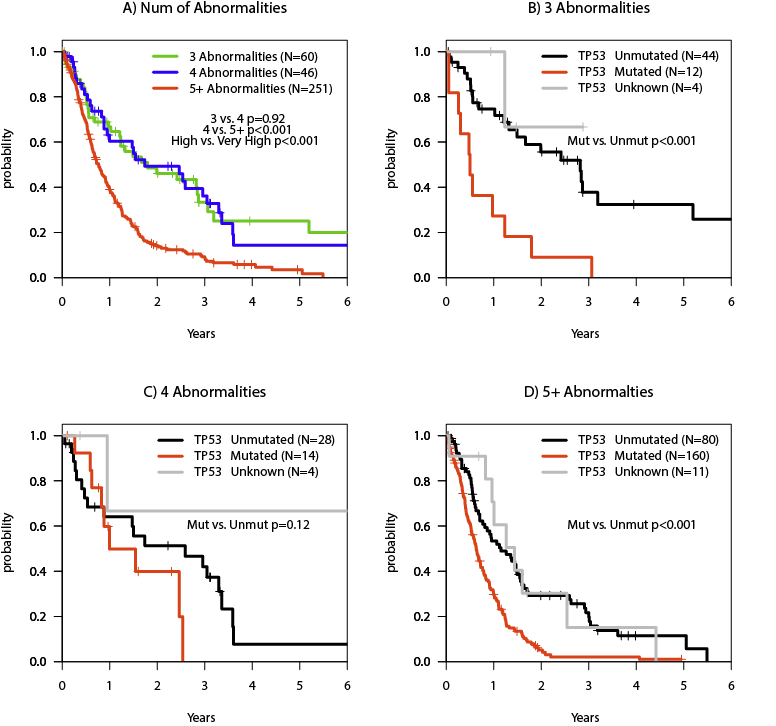


**Supplemental Figure 6:** Overall survival stratified by A) *SF3B1* and *TP53* mutation status and B) *NRAS* and *TP53* mutation status.


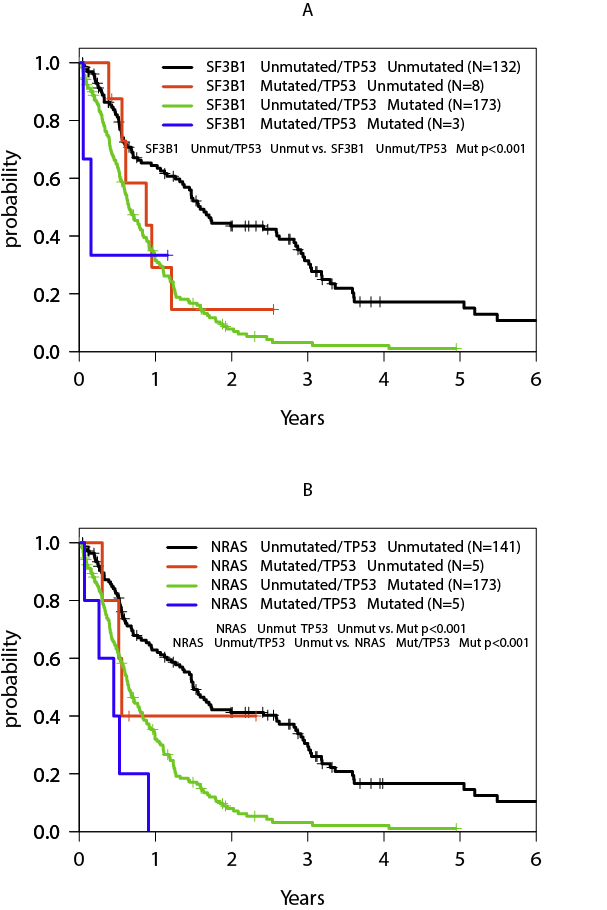


**Supplemental Figure 7:** Overall survival stratified by *TP53* mutation status and the presence of A) a chromosome 3 abnormality, B) a chromosome 9 abnormality, and C) monosomy 7.


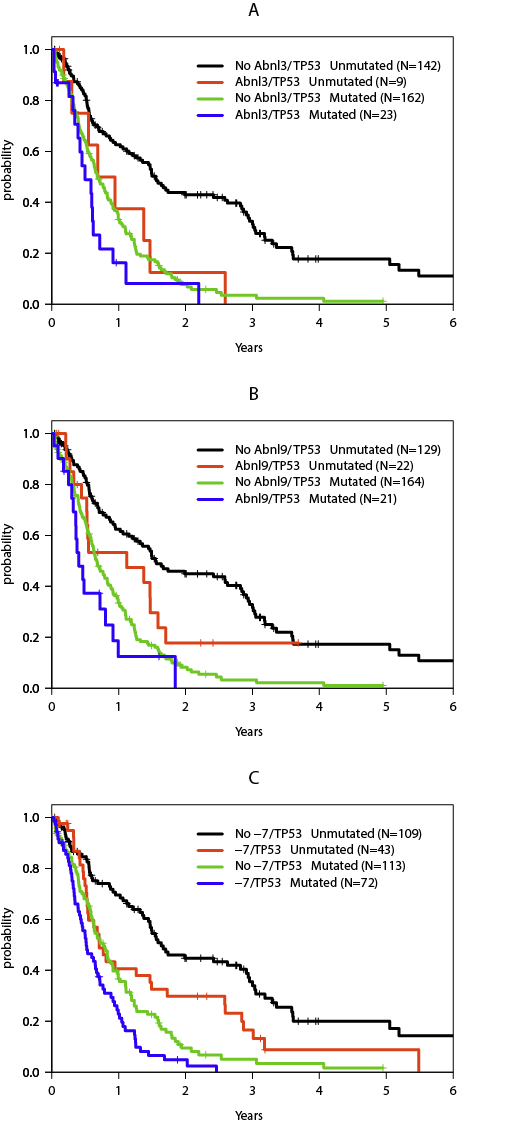


**Supplemental Figure 8:** Overall survival stratified by age, sex, bone marrow blast %, and peripheral blood counts.


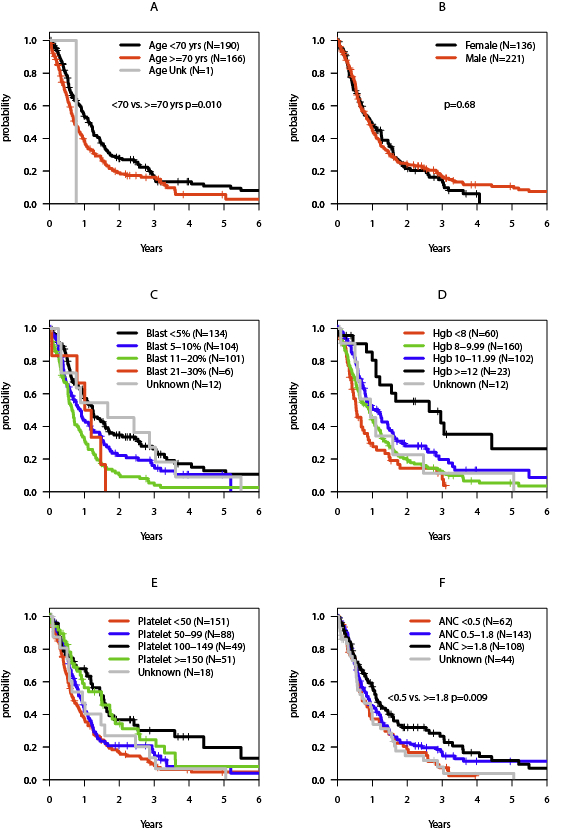


**Supplemental Figure 9:** Overall Survival by IPSS-R Risk Group and *TP53* Mutations Status


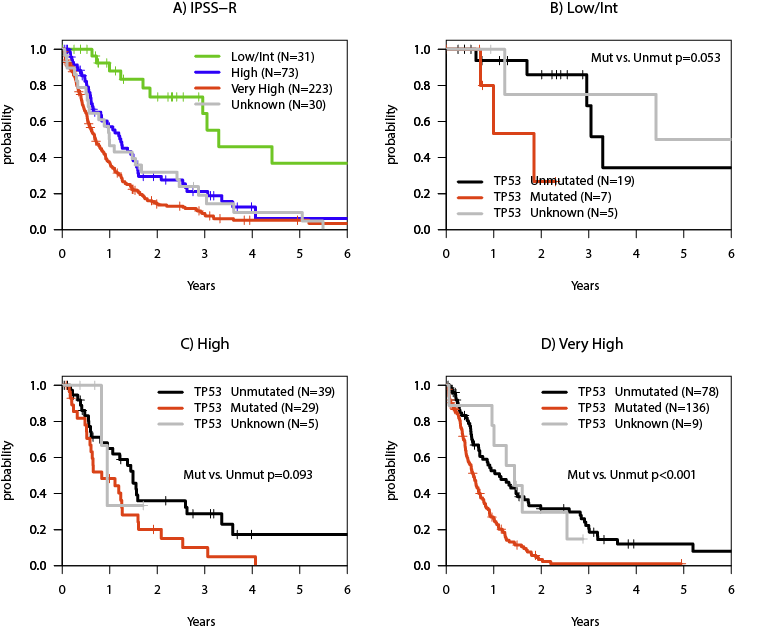


**Supplemental Figure 10:** Overall survival by A) t-MDS status, B) treatment status, and C) stem cell transplant status (landmarked analysis beginning 100 days post-transplant). Of the 16 patients known to have received a transplant, 4 were alive at 1.9, 3.1, 3.7, 8.1 years, and only one of these (known to survive until 1.9 years) had a *TP53* mutation (missense; p.R249S/c.747G>T; VAF=0.1785).


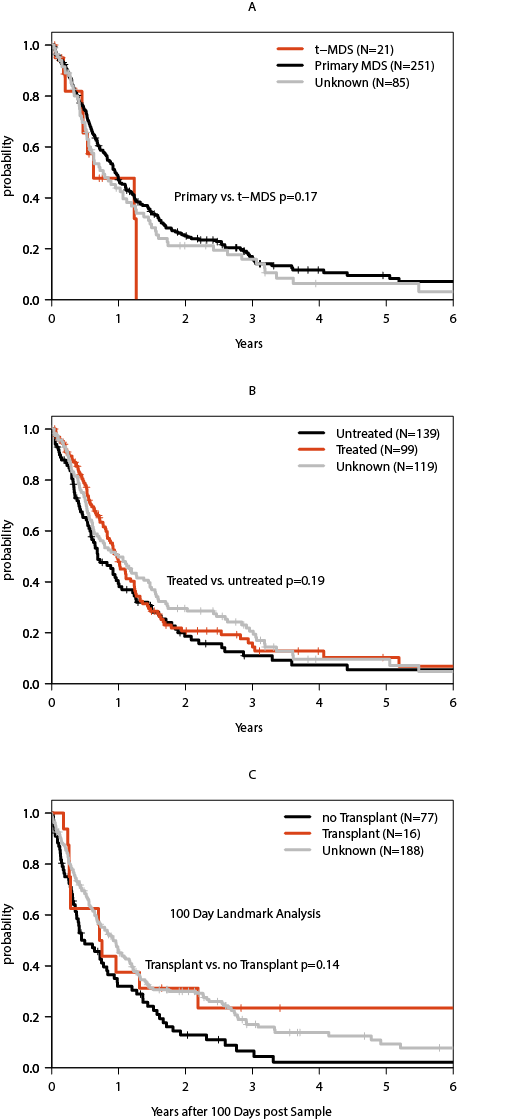

Supplement: Supplementary file 1 — Supplemental Tables and Figures [file 41375_2018_351_MOESM1_ESM.docx]
